# Supplementary material for: Biodegradation of highly crystallized poly(ethylene terephthalate) through cell surface codisplay of bacterial PETase and hydrophobin
Source: Nat Commun. 2022 Nov 21;13:7138. doi: 10.1038/s41467-022-34908-z (PMC9681837; doi:10.1038/s41467-022-34908-z)
Supplement: Supplementary file 1 — Supplementary Information [file 41467_2022_34908_MOESM1_ESM.pdf]

Supplementary Information For

Biodegradation of Highly Crystallized Poly(ethylene terephthalate) Through Cell Surface Codisplay of Bacterial PETase and Hydrophobin

Zhuozhi Chen [1, #], Rongdi Duan [1, #], Yunjie Xiao [1, #], Yi Wei [1, #], Hanxiao Zhang [1], Xinzhao Sun [1], Shen Wang [1], Yingying Cheng [1], Xue Wang [1], Shanwei Tong [1], Yunxiao Yao [1], Cheng Zhu[1], Haitao Yang [1, 2, 3], Yanyan Wang [1, *], and Zefang Wang [1, 2, *]

*Corresponding authors

Email: yanyanwang@tju.edu.cn (Yanyan W.); zefangwang@tju.edu.cn (Z.W.).

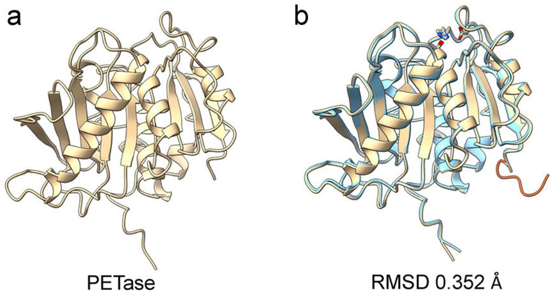

Supplementary Fig. 1 (a) The X-ray crystal structure of the native PETase. **(b)** Comparison of three-dimensional spatial structures of native PETase (yellow) and PETase-linker (cyan).

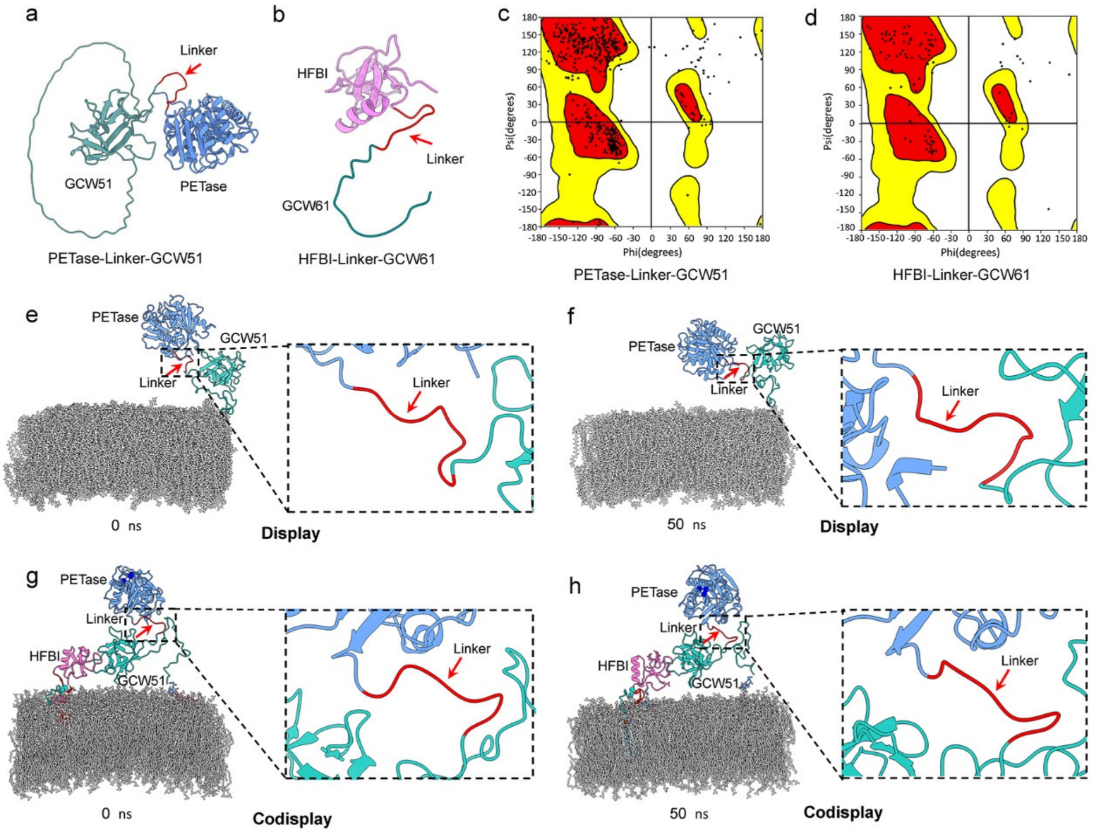

Supplementary Fig. 2 MD studies for the codisplayed PETase and HFBI. Model for PETase-Linker-GCW51 (a) and HFBI-Linker-GCW61 **(b)** were built with AlphaFold2. Each domain is assigned a unique color. The Ramachandran plot of the PETase-Linker-GCW51 **(c)** and HFBI-Linker-GCW61 **(d)** MD simulations of display system in 0 ns **(e)** and 50 ns **(f)** and Codisplay system in 0 ns **(g)** and 50 ns **(h)**. Each domain is assigned a unique color. The red arrow is marked as Linker.

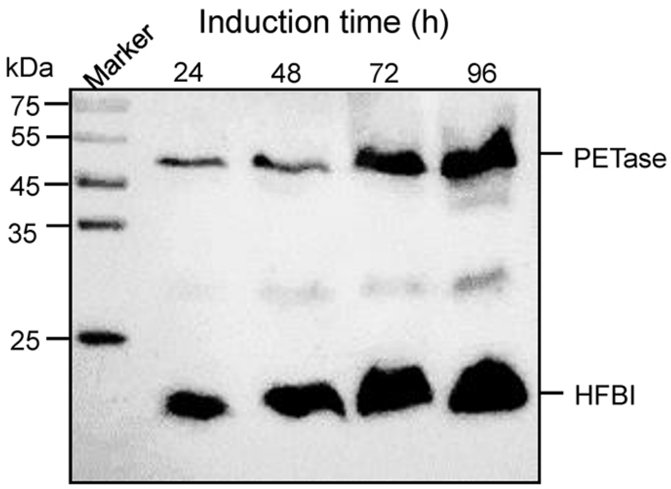

Supplementary Fig. 3 Western blot analysis of GS115/PETase-HFBI. The experiment is repeated three times independently with similar results obtained. One representative is shown. Source data is provided as a Source Data file.

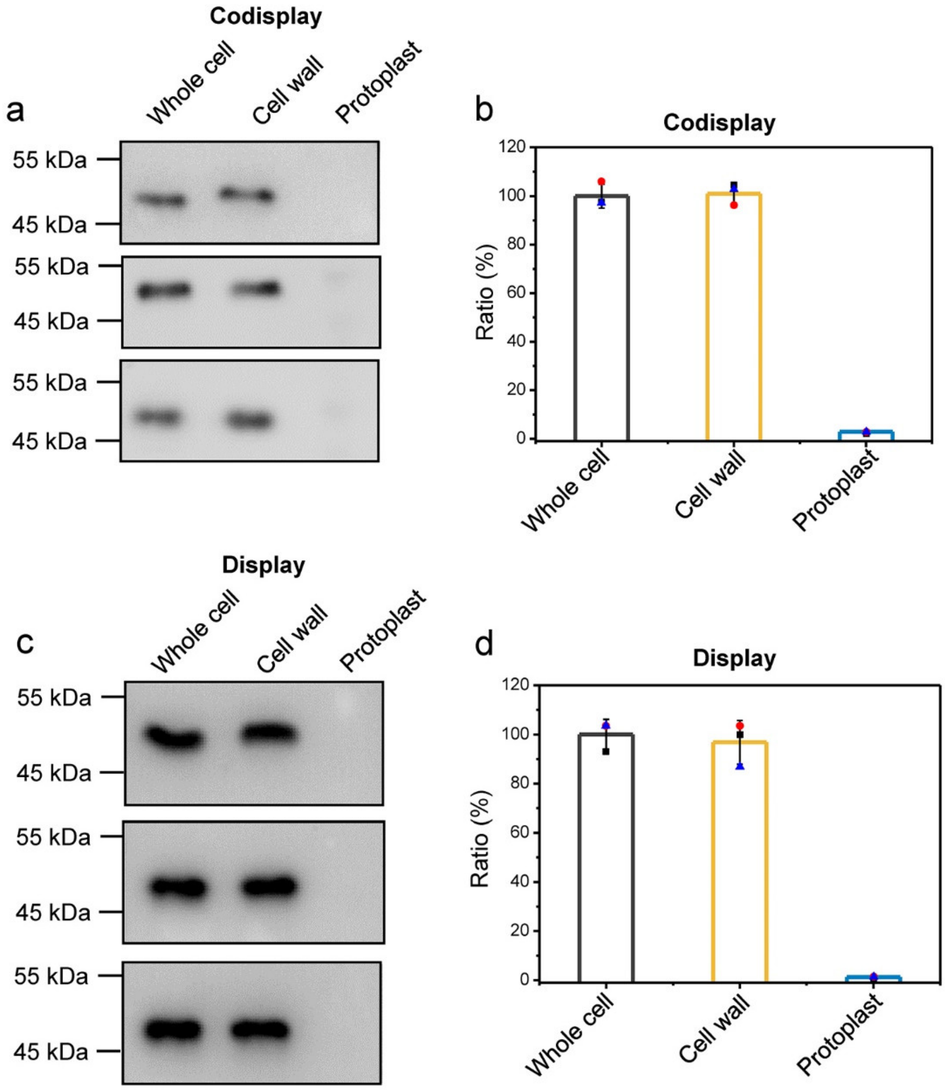

Supplementary Fig. 4 Western blot analysis of various components for *P. pastoris* GS115/PETase-HFBI and GS115/PETase-GCW51. (a) Western blot analysis of whole cell, cell wall and protoplast for GS115/PETase-HFBI. (Protein Marker, M221-10, GenStar, Beijing, China) **(b)** Measurement results of Western blot. **(c)** Western blot analysis of whole cell, cell wall and protoplast for GS115/PETase-GCW51. **(d)** Measurement results of Western blot. Data are presented as mean values +/- SD. The experiments are repeated three times independently with similar results obtained. One representative is shown. The samples derive from the same experiment and that gels/blots are processed in parallel. Source data are provided as a Source Data file.

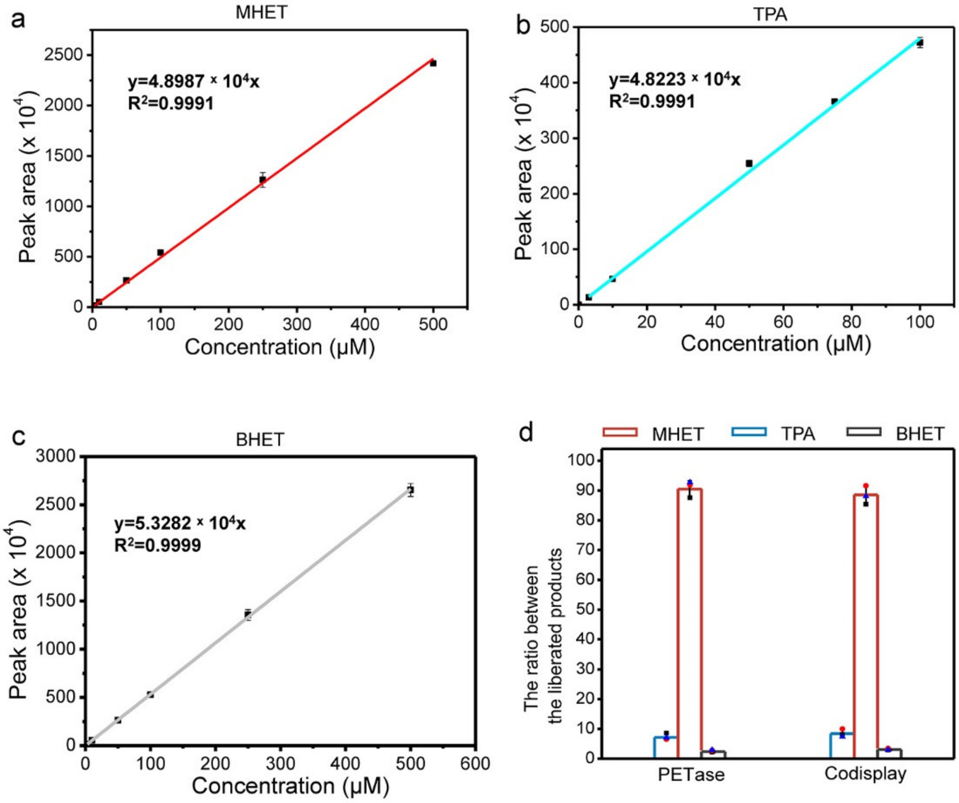

Supplementary Fig. 5 Standard curves of MHET **(a)** TPA **(b)** and BHET **(c)**. **(d)** The ratio between the liberated products of displayed PETase and that of the native PETase. All the experiments are repeated three times. Data are presented as mean values +/- SD. Source data are provided as a Source Data file.

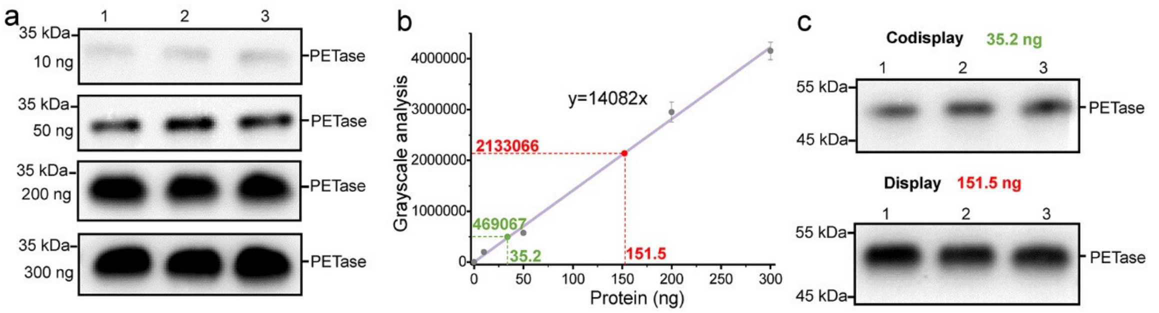

Supplementary Fig. 6 The normalized cell number to protein concentration for facilitate comparison. **(a)** The PETase-grayscale analysis about 10 ng, 50 ng, 200 ng and 300 ng by western blot. **(b)** The PETase-grayscale analysis standard curve. **(c)** PETase quantification was performed in 2×10^7 cells of *P. pastoris* GS115/PETase-HFBI and GS115/PETase-GCW51. All the experiments are repeated three times independently with similar results obtained. Data are presented as mean values +/- SD. The samples derive from the same experiment and that gels/blots are processed in parallel. Source data are provided as a Source Data file.

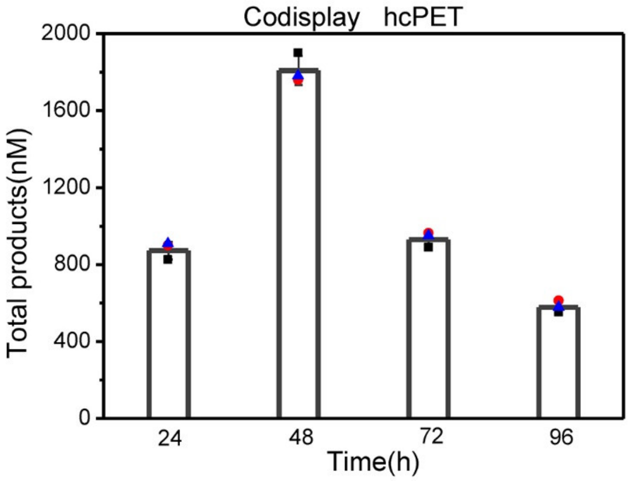

Supplementary Fig. 7 The total products measurement for the codisplay degradation hcPET system at different induction time. n=3 independent experiments. Data are presented as mean values +/- SD. Source data are provided as a Source Data file.

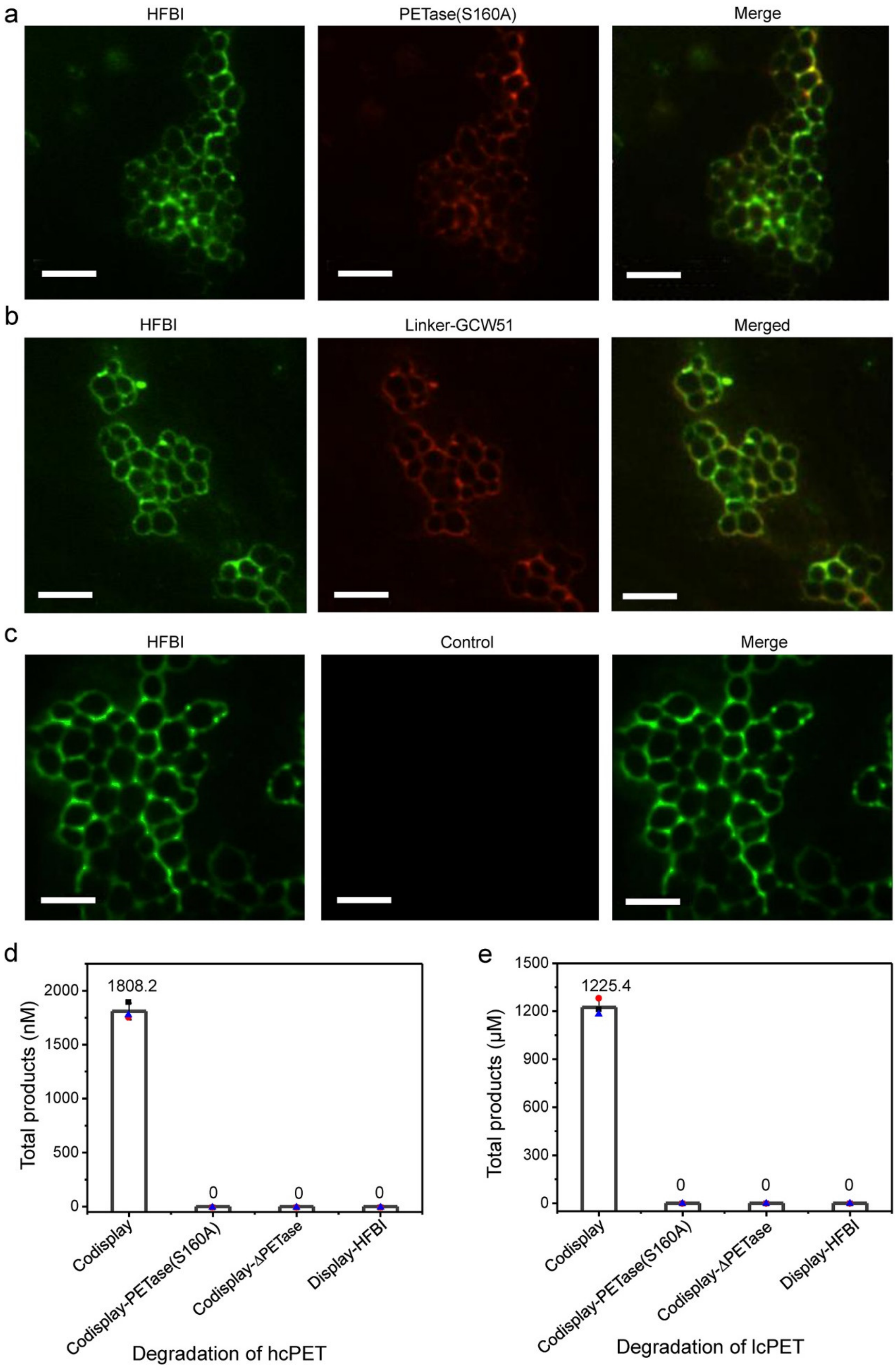

a
HFBI
PETase(S160A)
Merge
b
HFBI
Linker-GCW51
Merged
c
HFBI
Control
Merge
d
1808.2
0
0
0
Total products (nM)
2000
1500
1000
500
0
Codisplay
Codisplay-PETase(S160A)
Codisplay-ΔPETase
Display-HFBI
Degradation of hcPET
e
1225.4
0
0
0
Total products (μM)
1500
1200
900
600
300
0
Codisplay
Codisplay-PETase(S160A)
Codisplay-ΔPETase
Display-HFBI
Degradation of lcPET

Supplementary Fig. 8 Fluorescence microscopy of immuno-stained *P. pastoris* cells about **(a)** GS115/PETase(S160A)-HFBI, **(b)** GS115/ΔPETase-HFBI and **(c)** GS115/HFBI-GCW61. All the experiments are repeated three times independently with similar results obtained. One representative is shown for **(a)** to **(c)**. (Scale bar: 10 μm). The total products of hcPET **(d)** and lcPET **(e)** were degraded by the above three cells and GS115/PETase-HFBI. n=3 independent experiments. Data are presented as mean values +/- SD. Source data are provided as a Source Data file.

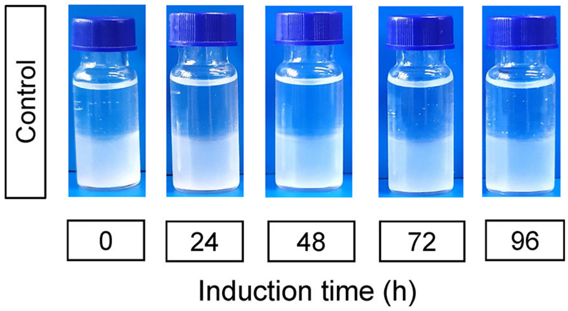

Supplementary Fig. 9 The real shot of MATH experiment yeast cells displaying only PETase. All the experiments are repeated three times independently with similar results obtained. One representative is shown.

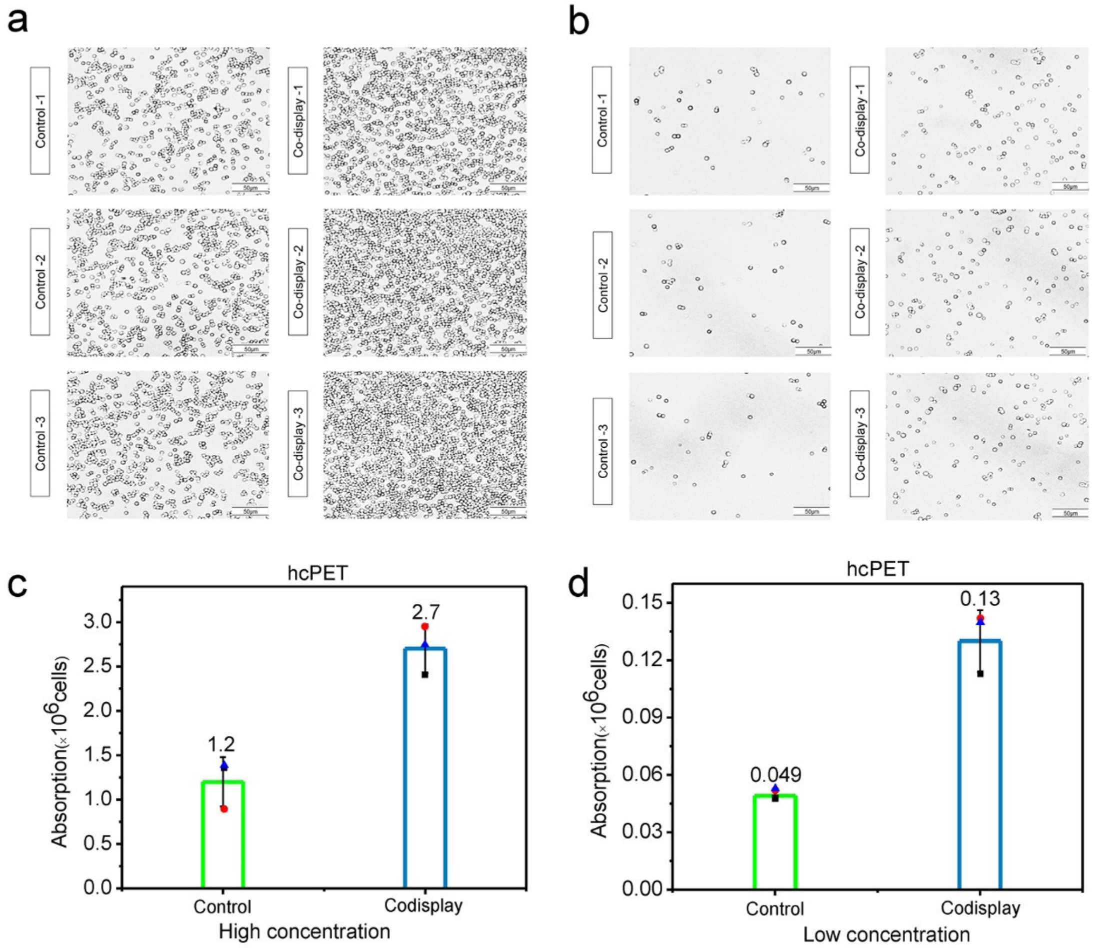

Supplementary Fig. 10 The adsorption of the control sample-yeast cells displaying only PETase and the codisplay cells on the hcPET surface **(a) & (c)** at high cell concentration; **(b) & (d)** at low cell concentration. (Scale bar: 50 μm). All the experiments are repeated three times independently with similar results obtained. One representative is shown. Data are presented as mean values +/- SD. Source data are provided as a Source Data file.

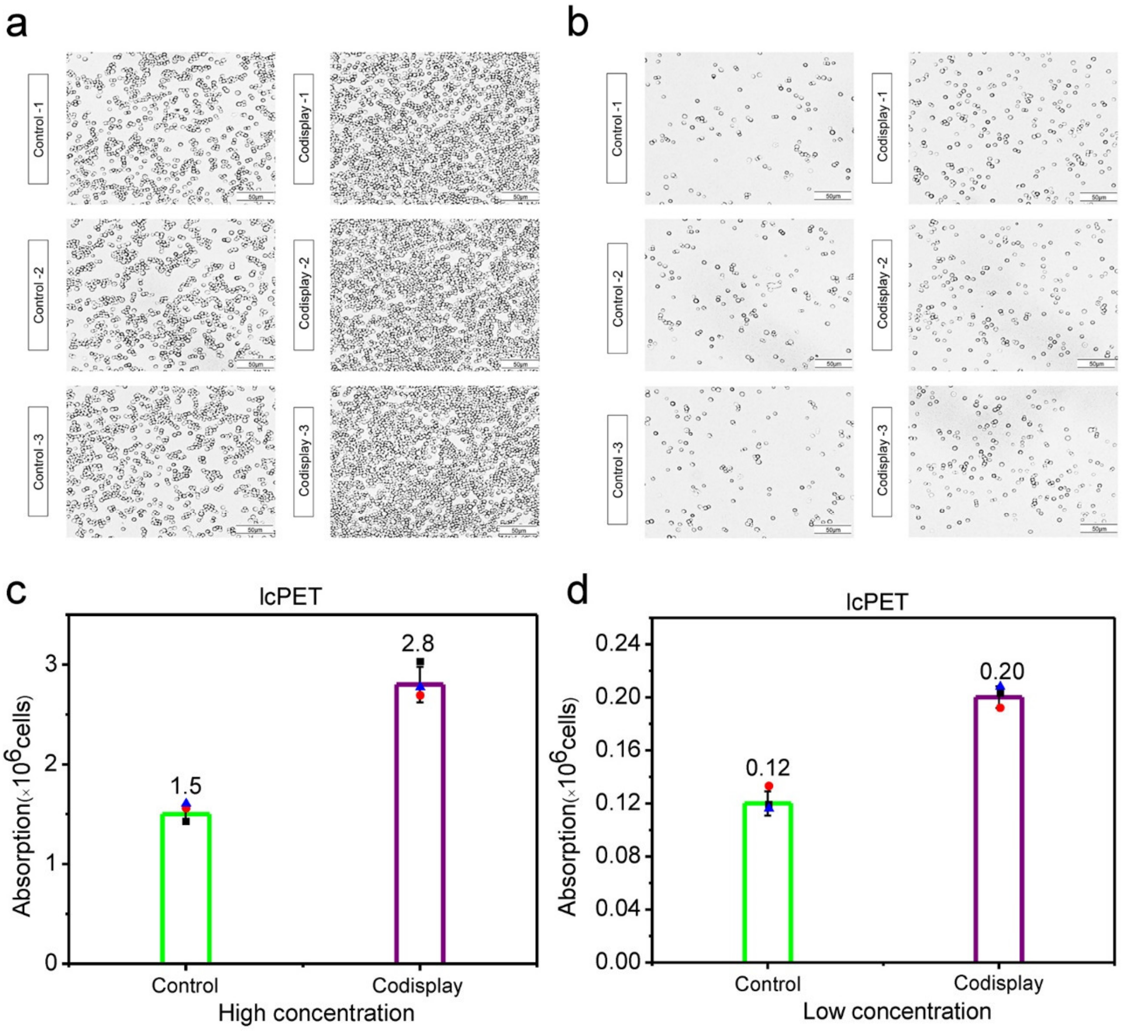

Supplementary Fig. 11 The adsorption of the control sample-yeast cells displaying only PETase and the codisplay cells on the lcPET surface **(a) & (c)** at high cell concentration; **(b) & (d)** at low cell concentration. (Scale bar: 50 μm). All the experiments are repeated three times independently with similar results obtained. One representative is shown. Data are presented as mean values +/- SD. Source data are provided as a Source Data file.

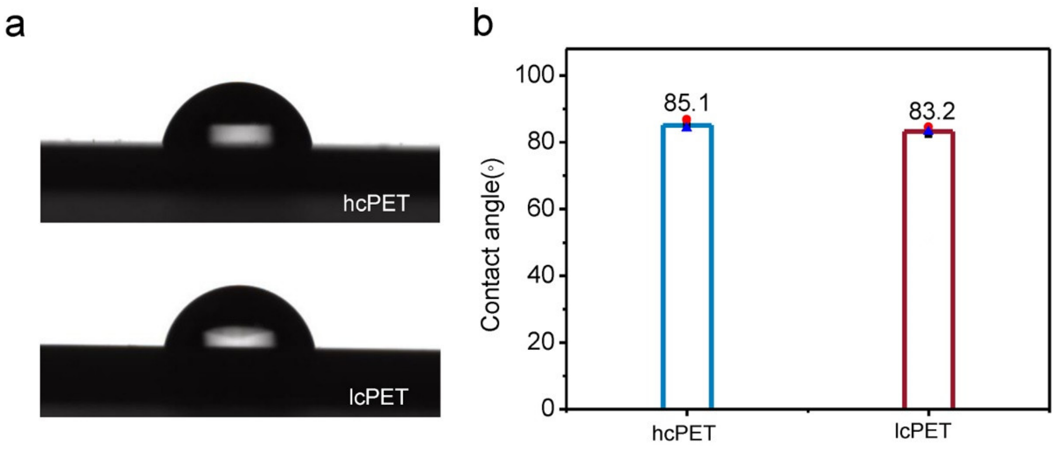

Supplementary Fig. 12 Comparison of the hydrophobicity of PET films. (a) WCA experiment on hcPET and lcPET to verify the hydrophobicity; **(b)** Measurement results of WCA. All the experiments are repeated three times independently with similar results obtained. One representative is shown. Data are presented as mean values +/- SD. Source data are provided as a Source Data file.

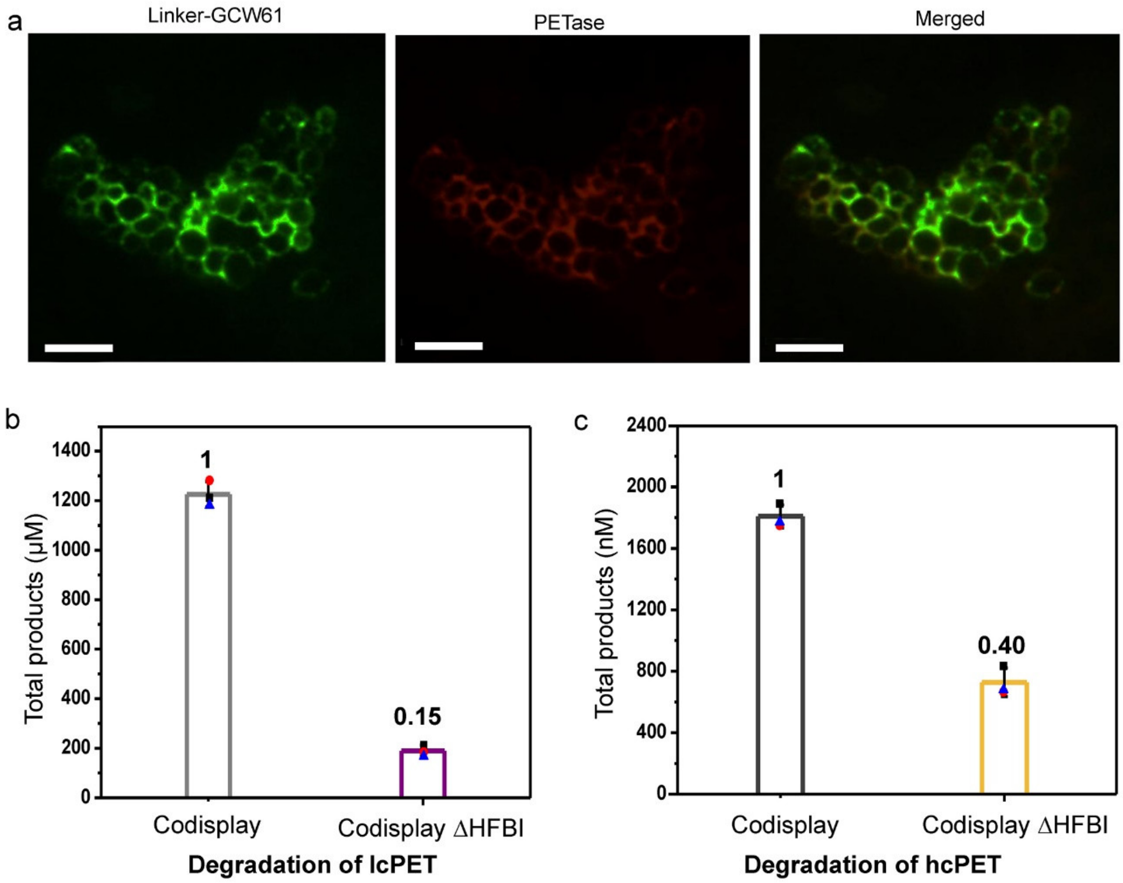

Supplementary Fig. 13 Enzymatic activity assay of negative control GS115/PETase-ΔHFBI cells. (a) Fluorescence microscopy of immuno-stained *P. pastoris* cells about GS115/PETase-ΔHFBI cells. (Scale bar: 10 μm). The total products of lcPET **(b)** and hcPET **(c)** were hydrolyzed by control sample GS115/PETase-HFBI cells and GS115/PETase-ΔHFBI cells. All the experiments are repeated three times independently with similar results obtained. One representative is shown. Data are presented as mean values +/- SD. Source data are provided as a Source Data file.

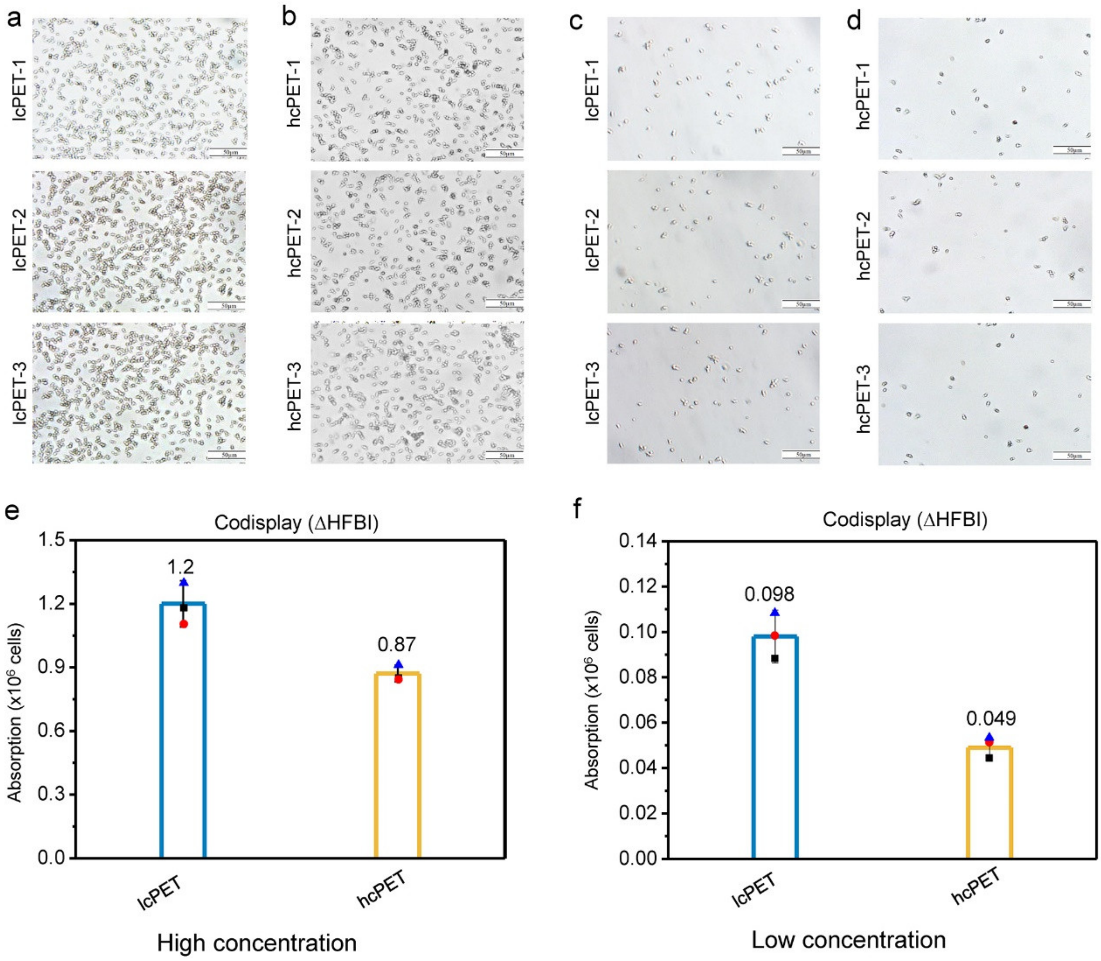

Supplementary Fig. 14 The adsorption of the GS115/PETase-ΔHFBI cells on the lcPET and hcPET surface at high cell concentration **(a) & (b) & (e)** and at low cell concentration **(c) & (d) & (f)**. (Scale bar: 50 μm). All the experiments are repeated three times independently with similar results obtained. One representative is shown. Data are presented as mean values +/- SD. Source data are provided as a Source Data file.

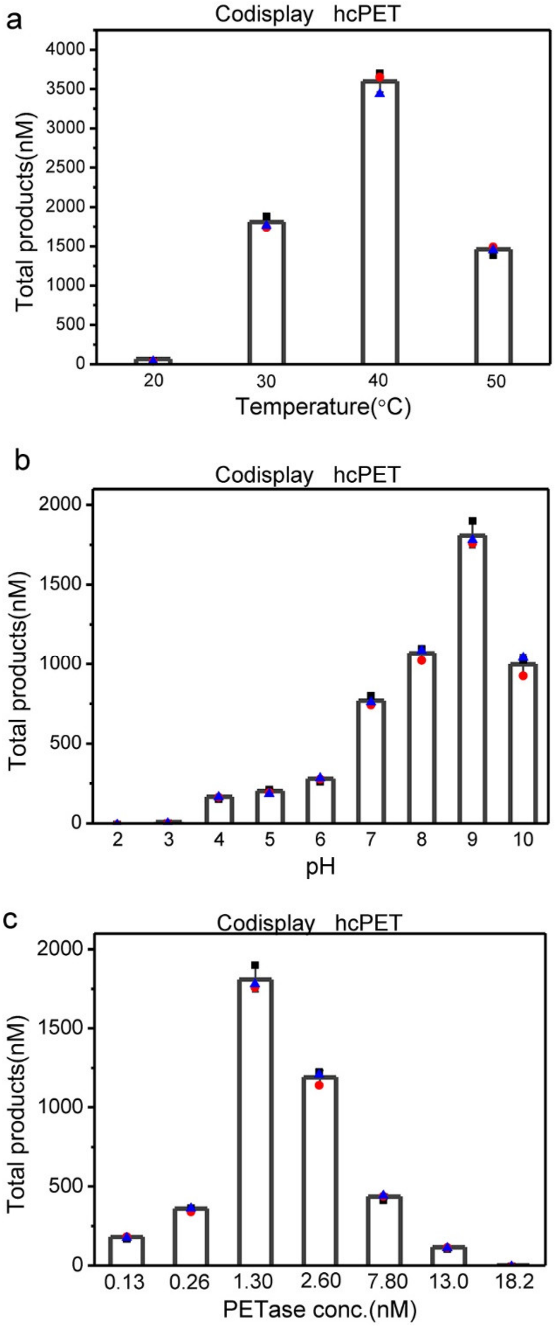

Supplementary Fig. 15 The total products measurement for the optimized codisplay degradation hcPET system. (a) Effect of temperature **(b)** pH and **(c)** protein concentration on PET hydrolysis. n=3 independent experiments. Data are presented as mean values +/- SD. Source data are provided as a Source Data file.

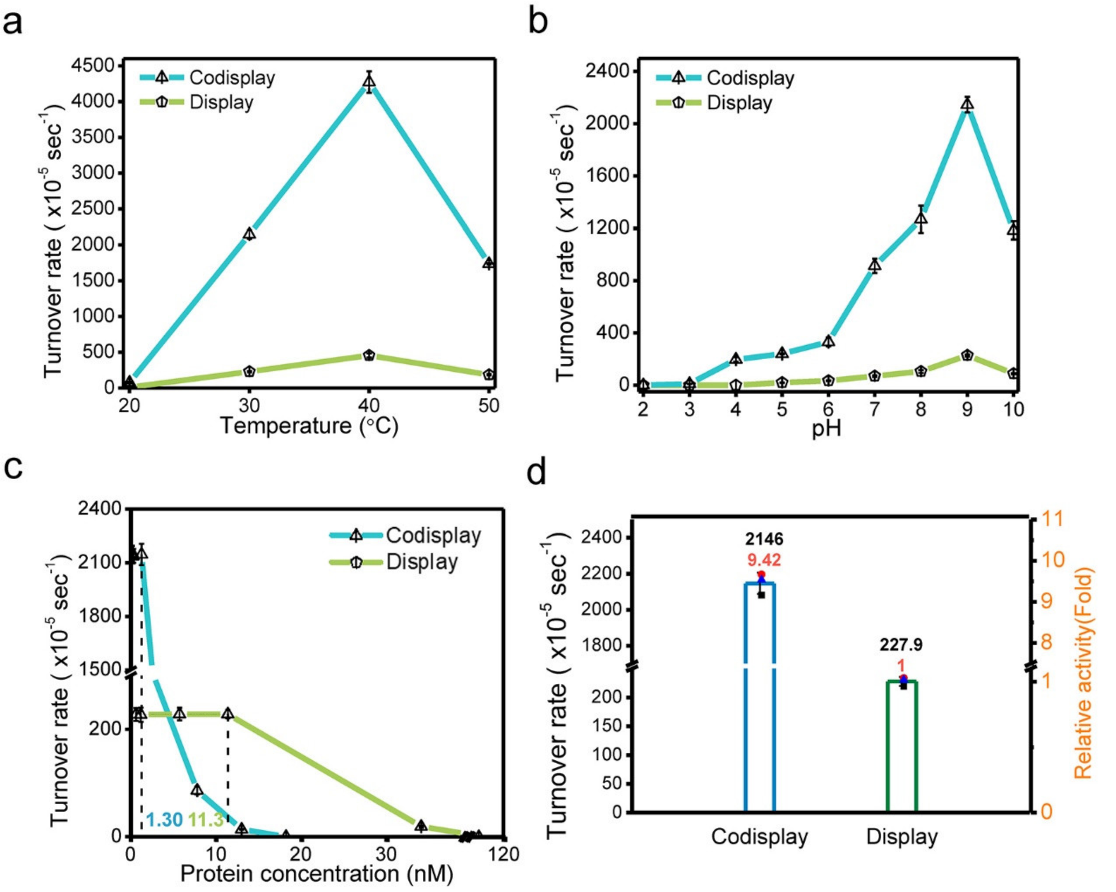

Supplementary Fig. 16 Comparison of display and co-display systems. (a) Effect of temperature **(b)** pH and **(c)** protein concentration on PET hydrolysis. **(d)** Comparison of the turnover rates of codisplay cells and display cells at the optimal condition using PET as a substrate. n=3 independent experiments. Data are presented as mean values +/- SD. Source data are provided as a Source Data file.

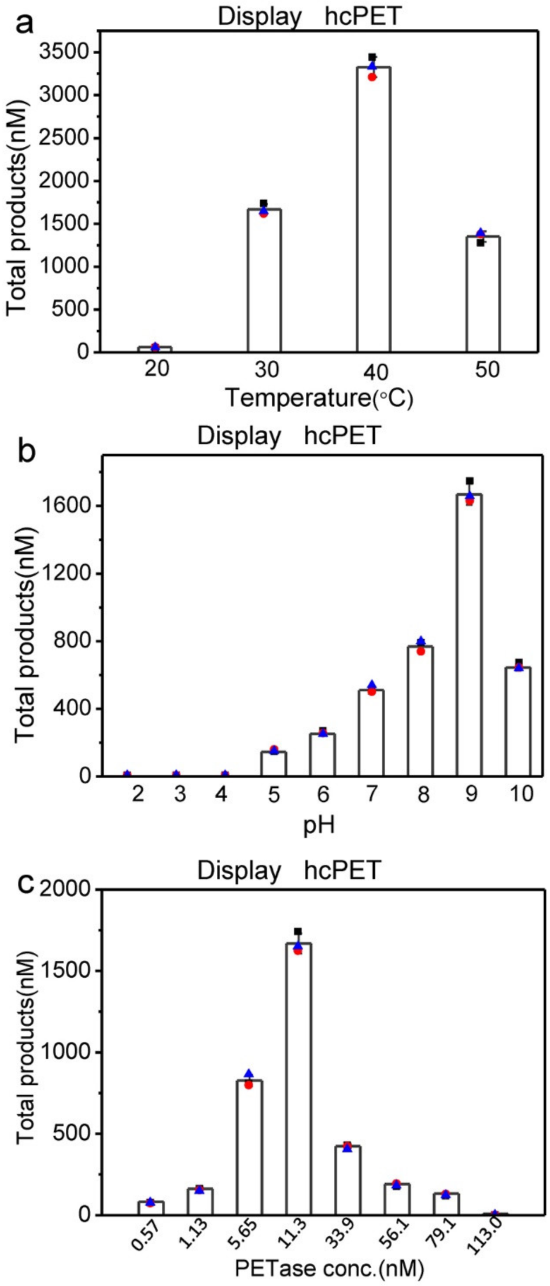

Supplementary Fig. 17 The total products measurement for the optimized display degradation hcPET system. (a) Effect of temperature **(b)** pH and **(c)** protein concentration on PET hydrolysis. n=3 independent experiments. Data are presented as mean values +/- SD. Source data are provided as a Source Data file.

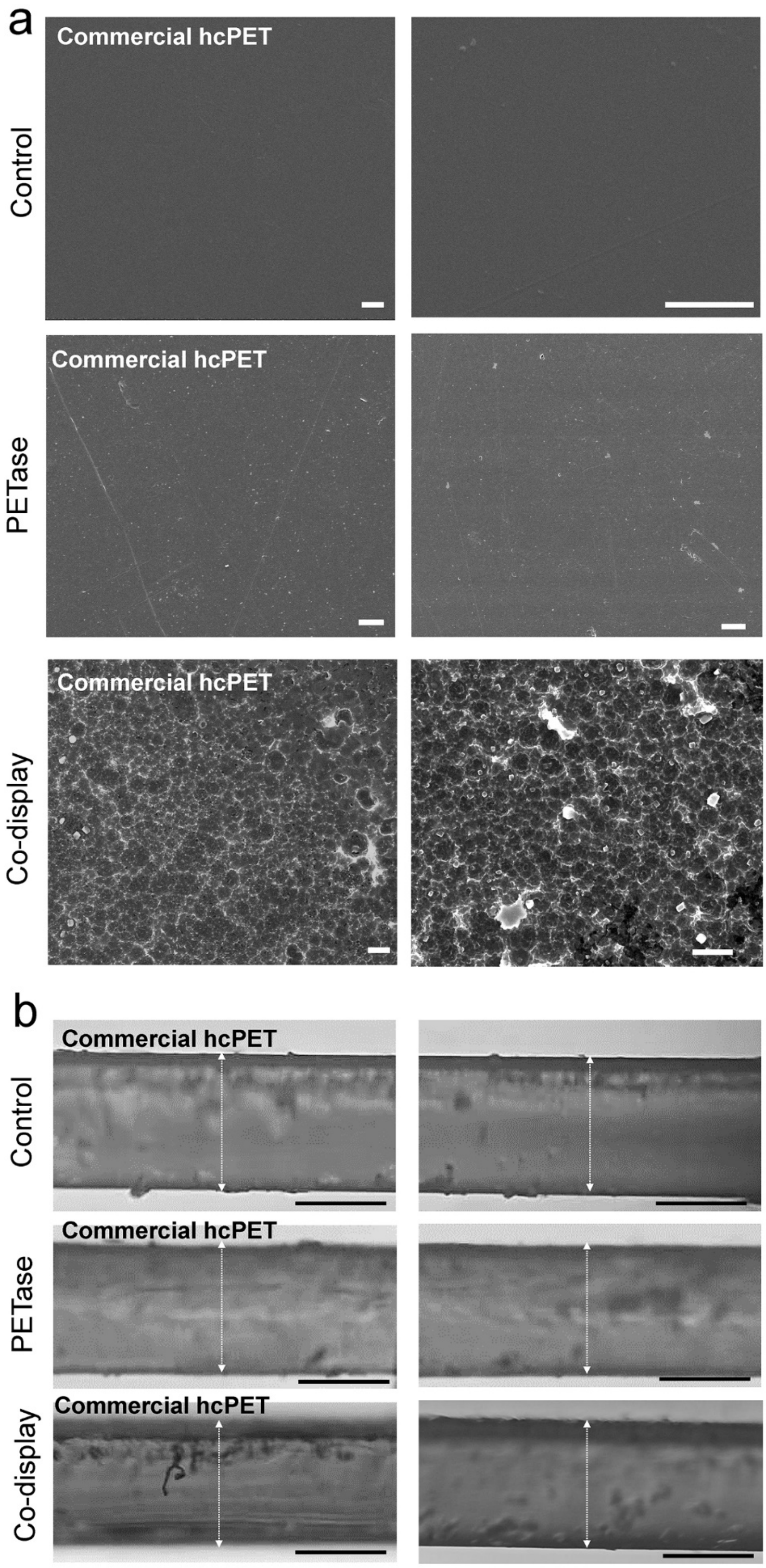
a
Commercial hcPET
Control
Commercial hcPET
PETase
Commercial hcPET
Co-display
b
Commercial hcPET
Control
Commercial hcPET
PETase
Commercial hcPET
Co-display

Supplementary Fig. 18 Visualization of hcPET film degradation. **(a)** SEM image of hcPET film before and after incubation with PETase and codisplay cells. (Scale bar: 5 μm) **(b)** Microscopic observation of cross-section of hcPET film before and after incubation with PETase and codisplay cells. (Scale bar: 100 μm). All the experiments are repeated three times independently with similar results obtained.

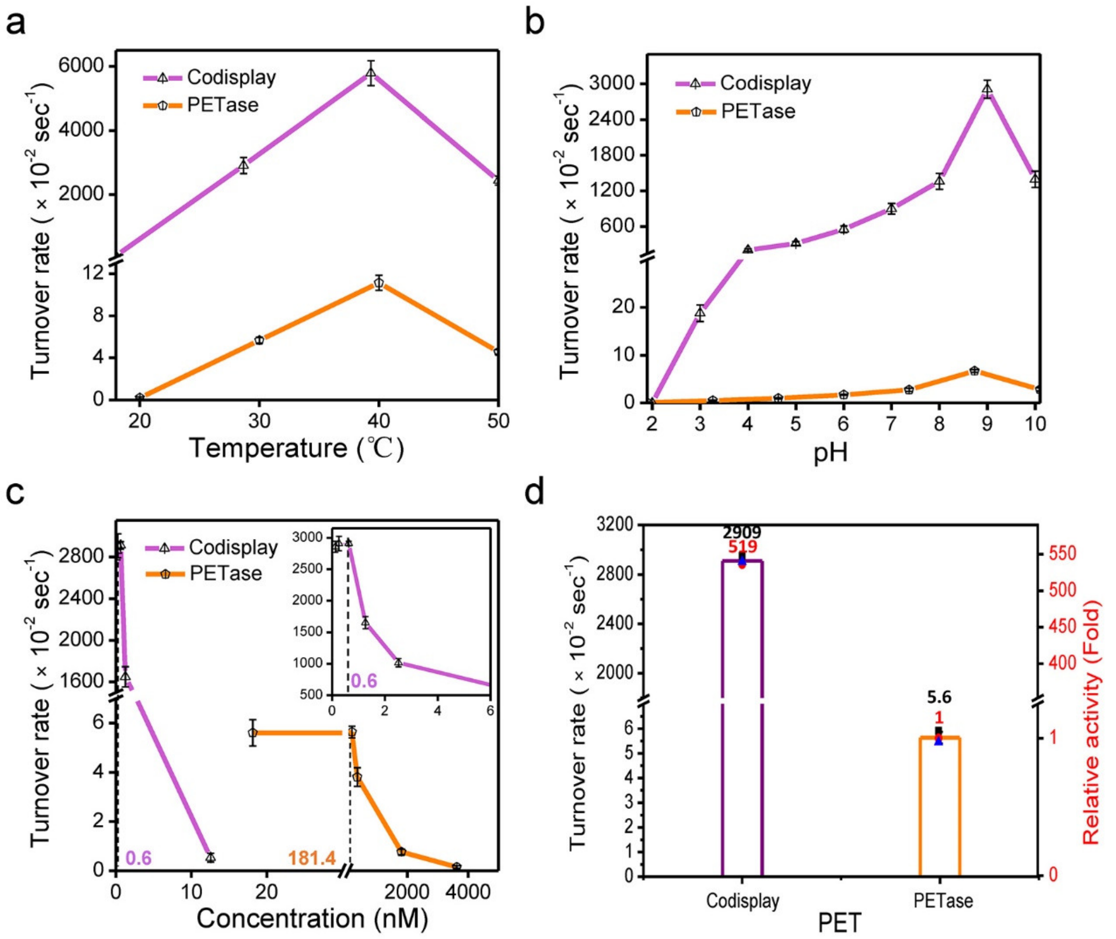

Supplementary Fig. 19 Optimization of the degradation system. (a) Effect of temperature **(b)** pH and **(c)** protein concentration on lcPET hydrolysis. **(d)** Comparison of the turnover rates of codisplay cells and PETase at the optimal condition using lcPET as substrate. n=3 independent experiments. Data are presented as mean values +/- SD. Source data are provided as a Source Data file.

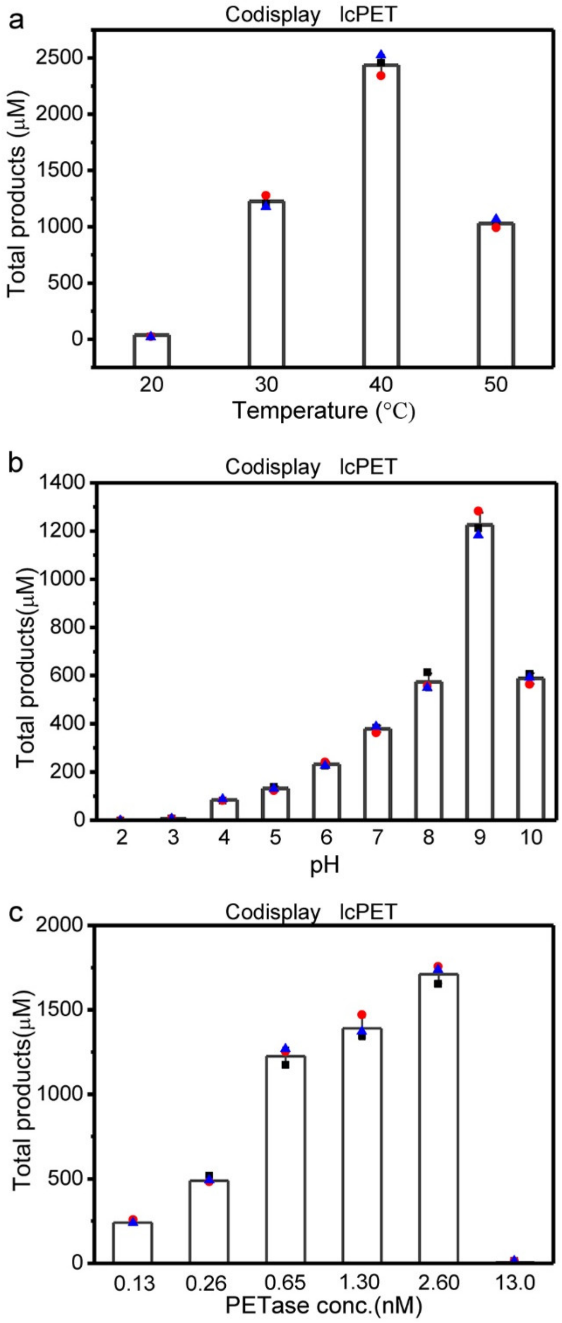

Supplementary Fig. 20 The total products measurement for the optimized codisplay degradation lcPET system. (a) Effect of temperature **(b)** pH and **(c)** protein concentration on PET hydrolysis. n=3 independent experiments. Data are presented as mean values +/- SD. Source data are provided as a Source Data file.

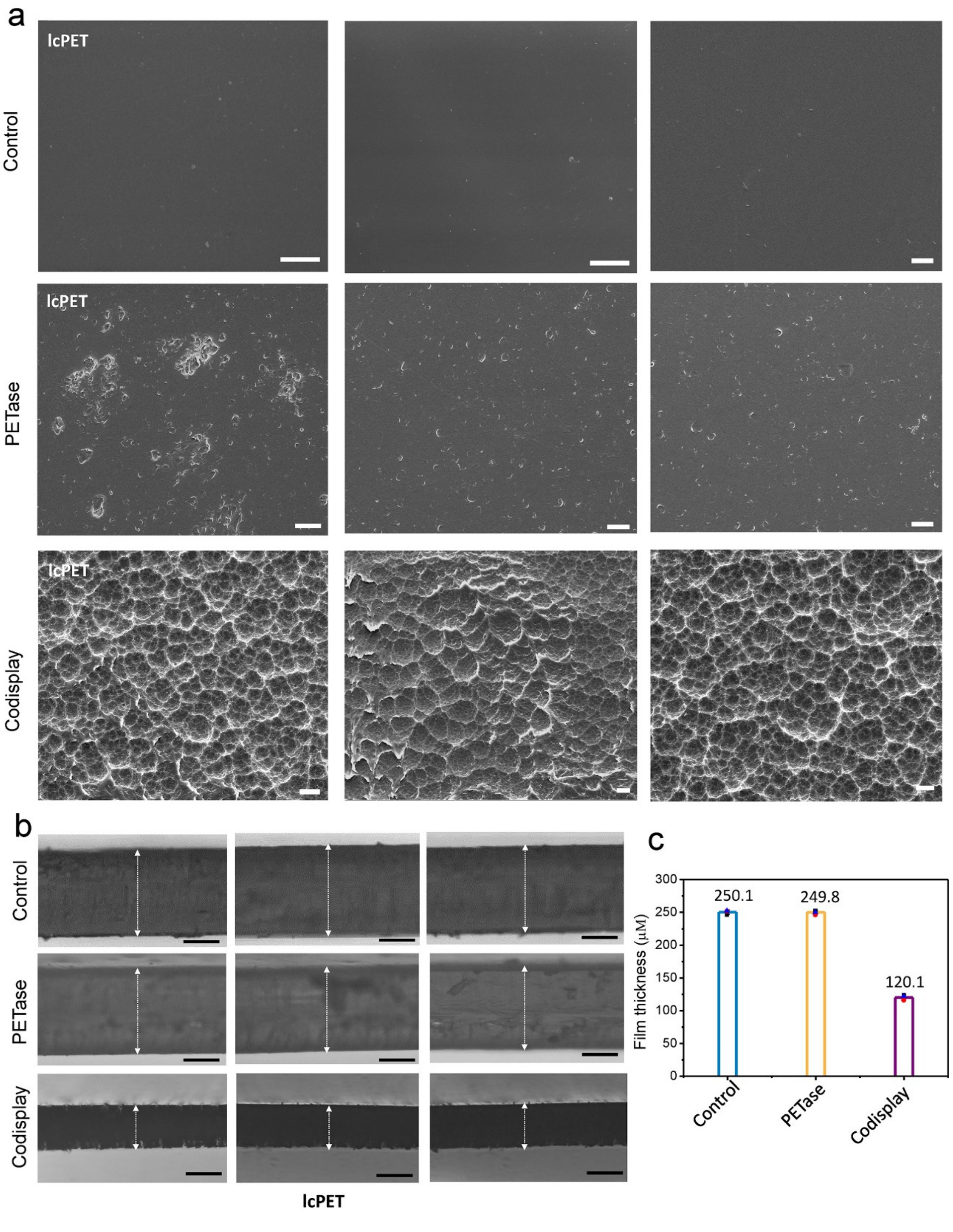

Supplementary Fig. 21 Visualization of lcPET film degradation. (a) SEM image of lcPET film before and after incubation with PETase and codisplay cells. (Scale bar: 5 μm) **(b)** Microscopic observation of cross-section of lcPET film before and after incubation with PETase and codisplay cells. (Scale bar: 100 μm). **(c)** Measurement of the cross-section. All the experiments are repeated three times independently with similar results obtained. Data are presented as mean values +/- SD. Source data are provided as a Source Data file.

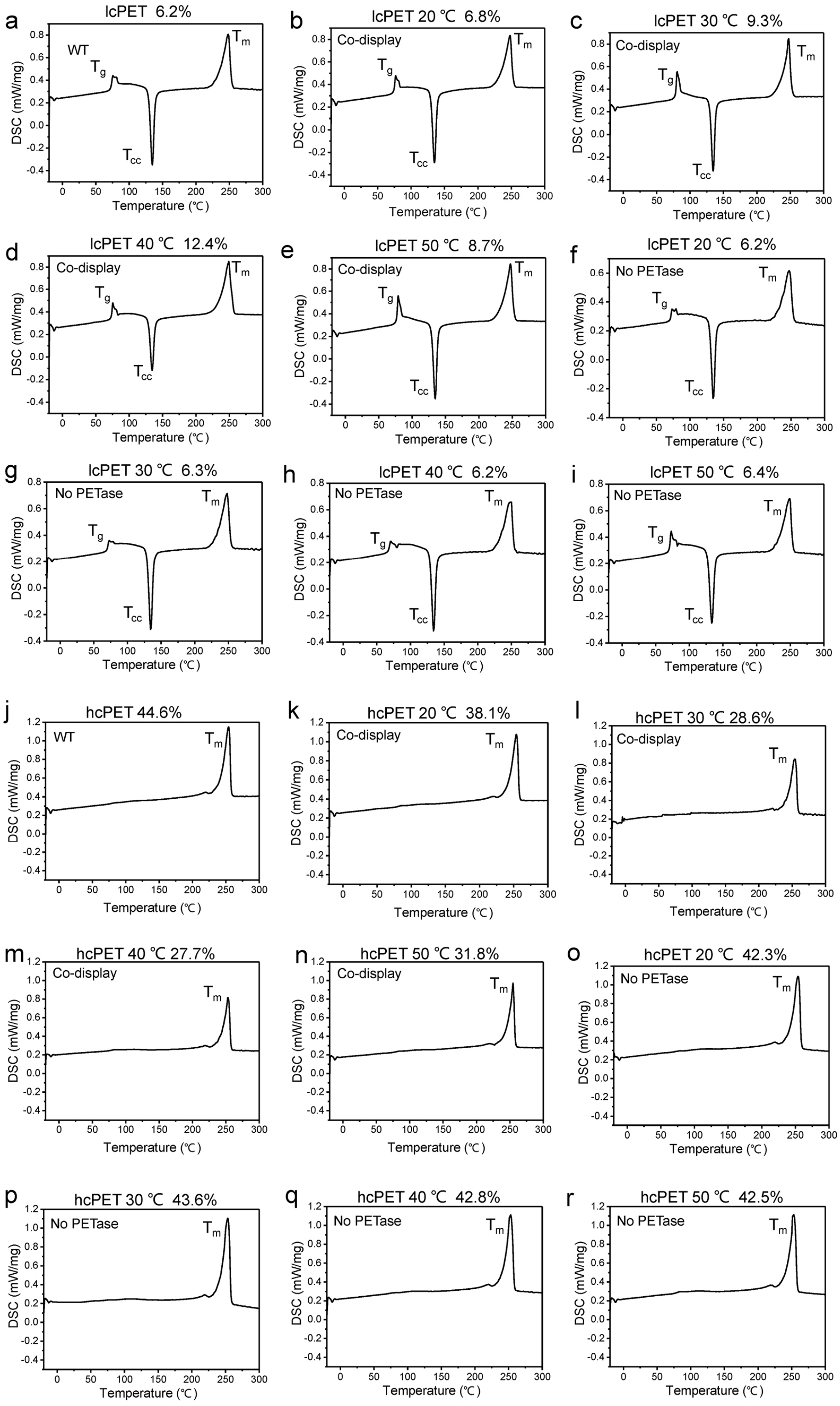

Supplementary Fig. 22 The crystallinity of lcPET **(a-i)** and hcPET **(j-r)** before and after degradation at different temperatures (20, 30, 40, and 50 °C) using the codisplay system. **(a)** The crystallinity of commercial lcPET. **(b-e)** The crystallinity of lcPET after degradation at different temperatures (20, 30, 40, and 50 °C) using the codisplay system. **(f-i)** The crystallinity of lcPET incubated at different temperatures

(20, 30, 40, and 50 °C). **(j)** The crystallinity of commercial hcPET. **(k-n)** The crystallinity of hcPET after degradation at different temperatures (20, 30, 40, and 50 °C) using the codisplay system. **(o-r)** The crystallinity of hcPET incubated at different temperatures (20, 30, 40, and 50 °C). All the experiments are repeated three times independently with similar results obtained. One representative is shown.

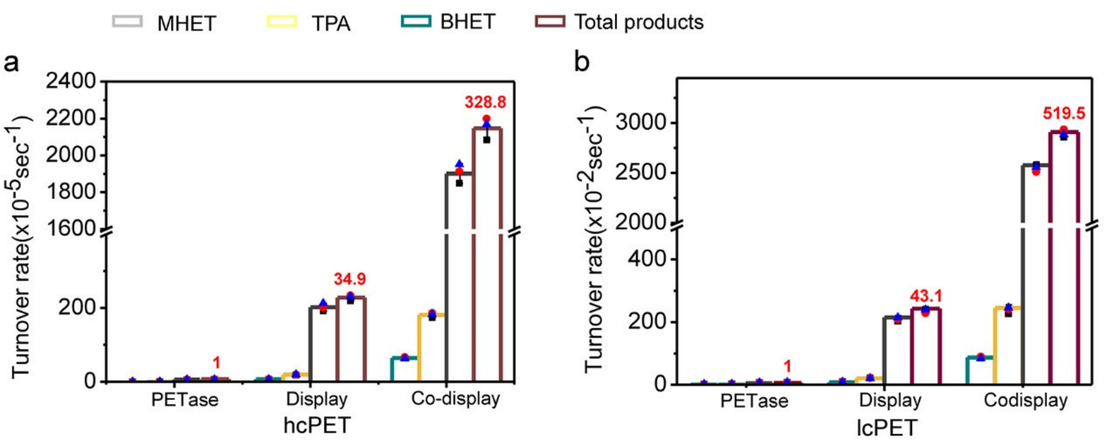

Supplementary Fig. 23 Comparison of the turnover rates based on the total products of MHET, TPA and BHET about purified PETase, display system and codisplay system at the optimal condition using lcPET **(a)** and hcPET **(b)** as substrate. n=3 independent experiments. Data are presented as mean values +/- SD. Source data are provided as a Source Data file.

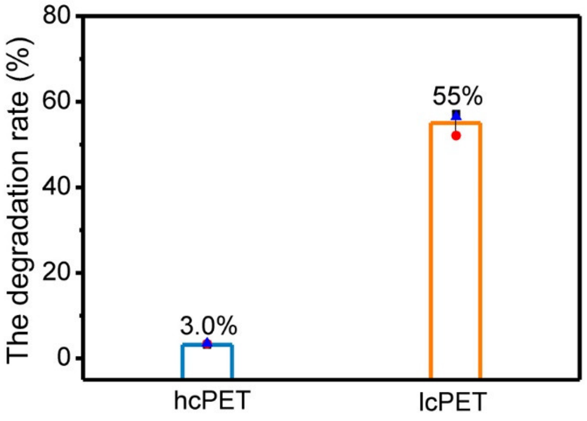

Supplementary Fig. 24 The degradation rates of hcPET and lcPET within 18 h at 30 °C were about 3.0% and 55%, respectively. n=3 independent experiments. Data are presented as mean values +/- SD. Source data are provided as a Source Data file.

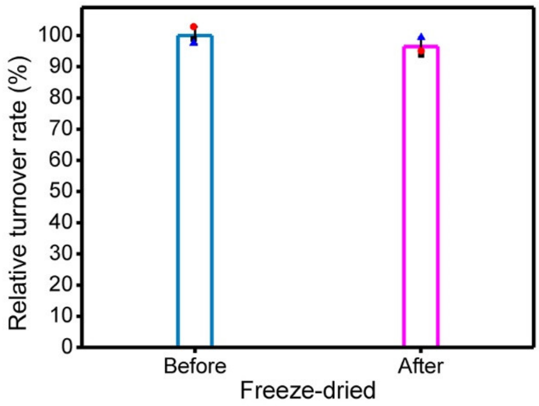

Supplementary Fig. 25 The hydrolysis of PET film with the codisplay system did not change much before and after freeze-drying. n=3 independent experiments. Data are presented as mean values +/- SD. Source data are provided as a Source Data file.

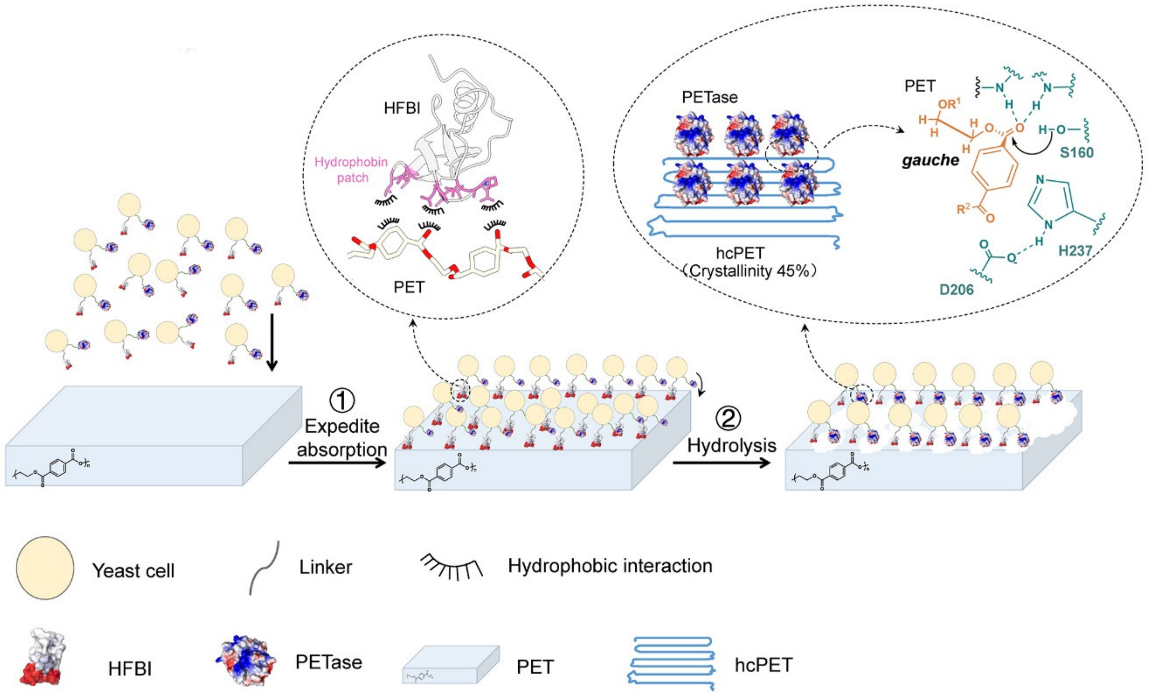

Supplementary Fig. 26 Schematic diagram of hydrolysis of hcPET by codisplay systerm. Firstly, due to the presence of HFBI, codisplayed cells quickly adsorbed to the hcPET surface, and the adsorption rate on the hcPET surface was close to 100%. Secondly, PETase contacts the surface of high crystallinity PET, and then hydrolyzes the PET chains, thus achieving the effect of efficient hydrolysis of high crystallinity PET. Different figures are used in the illustration to represent different elements.

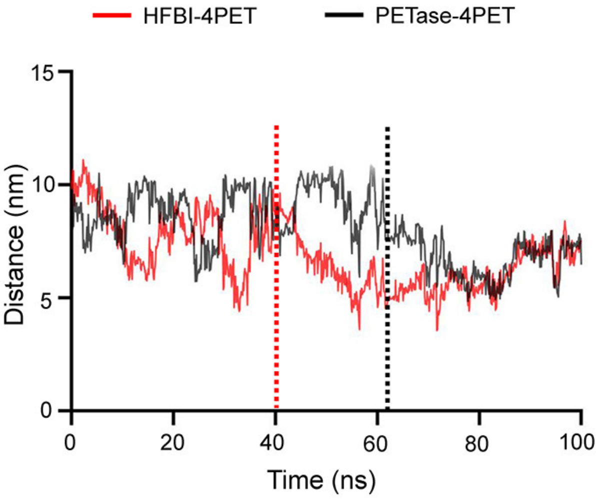

Supplementary Fig. 27 The change of distance between 20 4PET molecules and proteins (black line: PETase, red line: HFBI) in the GPI anchored system. The center of mass (COM) of each group were used in the gmx distance measurement. Around 40 ns, 4PET adhered to HFBI and remain mostly attached for the rest of the trajectory (red dash); at about 62 ns, 4PET came close to the catalytic triad of PETase (black dash).

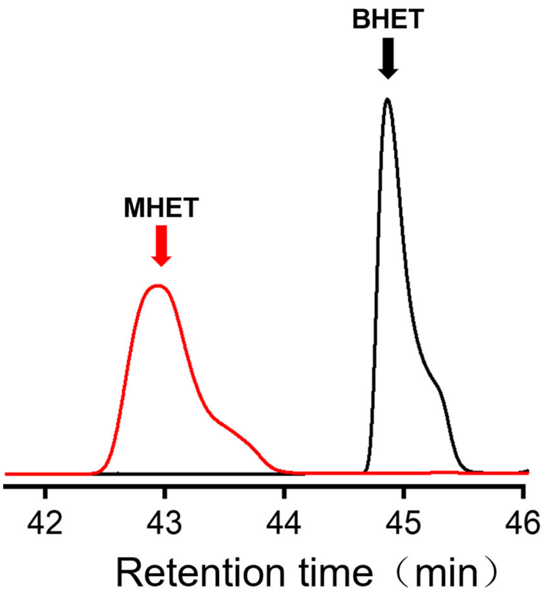

Supplementary Fig. 28 HPLC analysis of BHET before (black curve) and after hydrolysis. BHET is hydrolyzed to generate MHET (red curve). The experiments are repeated three times independently with similar results obtained. One representative is shown.

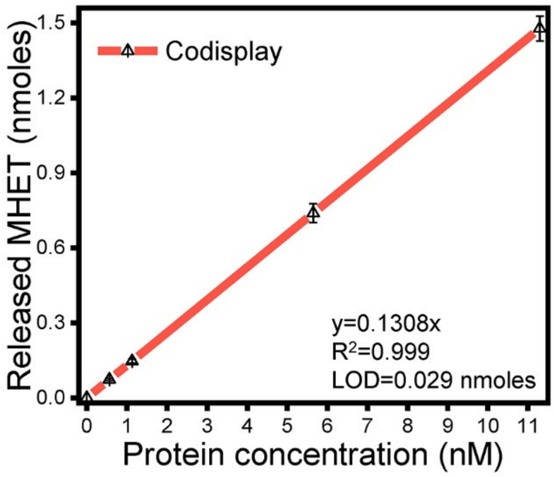

Supplementary Fig. 29 The LOD of HPLC analysis. n=3 independent experiments. Data are presented as mean values +/- SD. Source data are provided as a Source Data file.

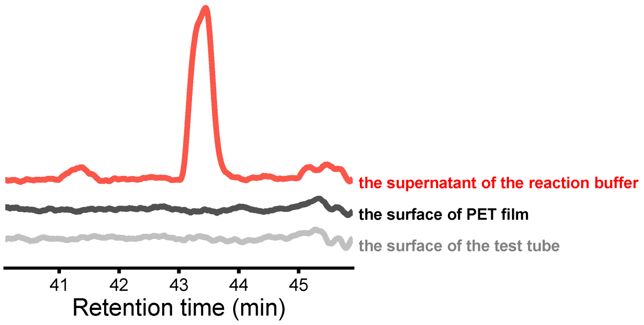

Supplementary Fig. 30 The HPLC measurements of three locations for the major degradation product MHET. The red curve is used to represent the supernatant of the reaction buffer. The black curve is used to represent the surface of the PET film. The grey curve is used to represent the surface of the test tube. The experiments are repeated three times independently with similar results obtained. One representative is shown.

Supplementary Sequence 1 The *petase* gene sequence

In this paper, the *petase* gene (GenBank number NZ_BBYR01000074.1) was first optimized and synthesized by BGI Group (China).

ATGCAAACCAATCCTTATGCCCGTGGTCCTAATCCTACCGCCGCGAGCTTAGA
AGCAAGCGCCGGTCCTTTTACCGTTCGTAGCTTTACCGTTAGCCGCCCATCAG
GTTATGGTGCCGGTACCGTTTACTATCCAACCAACGCCGGTGGTACCGTTGGT
GCCATTGCCATTGTTCCAGGTTACACCGCCCGTCAAAGCAGCATTAAGTGGTG
GGGTCCGCGTTTGGCCAGCCATGGTTTTGTTGTTATCACCATCGATACCAACA
GCACCTTGGATCAACCAAGCAGCCGTAGCAGCCAACAAATGGCCGCCTTGCG
TCAAGTTGCCAGCCTGAACGGTACTAGCAGCAGCCCAATTTACGGTAAGGTTG
ATACCGCCCGTATGGGTGTTATGGGTTGGAGCATGGGTGGTGGCGGTAGCTTG
ATTAGCGCCGCCAACAACCCAAGCTTGAAAGCCGCAGCACCACAAGCCCCAT
GGGATAGCAGCACCAACTTTAGCAGCGTTACCGTTCCTACCTTGATTTTTGCC
TGTGAGAACGATAGCATTGCCCCAGTTAACAGCAGCGCCTTGCCAATTTACGA
TAGCATGAGCCGTAACGCCAAGCAATTCTTAGAAATCAACGGTGGTAGCCAT
AGCTGTGCCAACAGCGGTAACAGCAACCAAGCCTTGATTGGTAAGAAAGGCG
TTGCCTGGATGAAGCGTTTTATGGATAACGATACCCGTTACAGCACCTTTGCC
TGTGAAAACCCAAACAGCACCCGTGTTAGCGATTTTCGTACCGCCAACTGTAG
CTGA

Supplementary Table 1. X-ray data collection and refinement statistics

	PETase	PETase-linker
**Data collection**		
Space group	$P2_12_12_1$	$P2_12_12_1$
Cell dimensions		
a, b, c (Å)	51.4, 55.5, 85.5	50.8, 59.9, 77.4
α, β, γ (°)	90, 90, 90	90, 90, 90
Wavelength (Å)	0.97861	0.97864
Resolution limit (Å)	50.00-2.00 (2.03-2.00)*	50.00-1.50 (1.54-1.50)*
*R*merge (%)	17.2 (>100)	19.4 (>100)
$I / \sigma I$	18.1 (2.9)	14.1 (4.1)
Completeness (%)	99.7 (99.9)	98.6 (99.6)
Redundancy	5.5 (5.5)	12.2 (9.7)
**Refinement**		
Resolution range (Å)	50.00-2.00	50.00-1.50
No. reflections	16369 (1423)	37916(3634)
R_{work} / R_{free}	0.180/0.219	0.159/0.190
NO. atoms		
Protein	1934	1959
Ligand/ion	0	0
Water	159	393
B-factors		
Protein	30.22	12.62
Ligand/ion	/	/
Water	37.80	27.13
**R.m.s deviations**		
Bond lengths (Å)	0.009	0.009
**Ramachandran statistics (%)**		
Favored	96.93	98.13
Allowed	3.07	1.87
Outliers	0.00	0.00

Supplementary Table 2 The yeast strains and plasmids used in this study

Plasmids/yeast strains	Description
**Plasmids**	
pPIC9	The yeast expression vector for cell surface display
pPICZαA	The yeast expression vector for cell surface display
pPIC9-PETase-GCW51	PETase and GCW51 fusion protein gene sequence expressed in pPIC9
pPICZαA-HFBI-GCW61	HFBI and GCW61 fusion protein gene sequence expressed in pPICZαA
pPICZαA-linker-GCW61	Linker and GCW61 fusion protein gene sequence expressed in pPICZαA
**Strains**	
P. pastoris GS115	The methylotrophic yeast, capable of metabolizing methanol as individual sole carbon source; a mutation in the histidine dehydrogenase gene (HIS4); histidine auxotroph
P. pastoris GS115/PETase-GCW51	GS115 strain harboring pPIC9-PETase-GCW51
P. pastoris GS115/PETase-HFBI	GS115 strain harboring pPIC9-PETase-GCW51 and pPICZαA-HFBI-GCW61
P. pastoris GS115/PETase-ΔHFBI	GS115 strain harboring pPIC9-PETase-GCW51 and pPICZαA-linker-GCW61
P. pastoris GS115/ΔPETase-HFBI	GS115 strain harboring pPIC9-linker-GCW51 and pPICZαA-HFBI-GCW61
P. pastoris GS115/PETase(S160A)-HFBI	GS115 strain harboring pPIC9-PETase(S160A)-GCW51 and pPICZαA-HFBI-GCW61
P. pastoris GS115/HFBI-GCW61	GS115 strain harboring pPICZαA-linker-GCW61

Supplementary Table 3 The primers used for plasmid construction

Primers	Sequences
51-F	GGTGGTGGAGGTAGTGGAGGAGGTGGTAGTGATGACGATGACTCATTAC
51-R	CCGGAATTCCTAGATCAATAGGGCAATGG
61-F	GGTGGTGGAGGTAGTGGAGGAGGTGGTAGTAACAACCTATCAAACGAGAGT
61-R	ATAGTTTAGCGGCCGCTTAAATCAATAGAGCAACACCGGC
P-F	CCGCTCGAGAAAAGAGATTACAAGGATGACGACGATAAGCAAACCAATCCTTATGCCCGTG
P-R	ACTACCACCTCCTCCACTACCTCCACCACCGCTACAGTTGGCGGTACGAA
hf-F	CCGGAATTCCAACAGTGCACCACTGG
hf-R	ACTACCACCTCCTCCACTACCTCCACCACCGACGTTAACCGGAACACATC
ΔHFBI-F	CCGGAATTCCAT CAT CAC CAT CAC CATGGTGGTGGAGGTAGTGGAGG
ΔHFBI-F	ATAAGAATGCGGCCGCTTAAATCAATAGAGCAACACCG
ΔPETase-F	CCGCTCGAGAAAAGAGATTACAAGGATGACGACGATAAGGGTGGTGGAGGTAGTGGAGG
ΔPETase-R	CCGGAATTCCTAGATCAATAGGGCAATGGC
p51-F	GGAATTCCATATGCAAACCAATCCTTATGCCCG
p51-R	CCGCTCGAGCTAGATCAATAGGGCAATGGCAAC
PL-F	GGAATTCCATATGCAAACCAATCCTTATGCCCG
PL-R	CCGCTCGAGACTACCACCTCCTCCACTACCTCCA
PETase (S160A)-F	CCGCTCGAGAAAAGAGATTACAAGGATGACG
PETase (S160A) -overlap-F	GTTATGGGTTGGGCCATGGGTGGTGG
PETase (S160A) -overlap-R	CCACCACCCATGGCCCAACCCATAAC
PETase (S160A)-R	CCGGAATTCCTAGATCAATAGGGCAATGG

Primers	Sequences
51-F	GGTGGTGGAGGTAGTGGAGGAGGTGGTAGTGATGACGATGACTCATTAC
51-R	CCGGAATTCCTAGATCAATAGGGCAATGG
61-F	GGTGGTGGAGGTAGTGGAGGAGGTGGTAGTAACAACCTATCAAACGAGAGT
61-R	ATAGTTTAGCGGCCGCTTAAATCAATAGAGCAACACCGGC
P-F	CCGCTCGAGAAAAGAGATTACAAGGATGACGACGATAAGCAAACCAATCCTTATGCCCGTG
P-R	ACTACCACCTCCTCCACTACCTCCACCACCGCTACAGTTGGCGGTACGAA
hf-F	CCGGAATTCCAACAGTGCACCACTGG
hf-R	ACTACCACCTCCTCCACTACCTCCACCACCGACGTTAACCGGAACACATC
ΔHFBI-F	CCGGAATTCCAT CAT CAC CAT CAC CATGGTGGTGGAGGTAGTGGAGG
ΔHFBI-F	ATAAGAATGCGGCCGCTTAAATCAATAGAGCAACACCG
ΔPETase-F	CCGCTCGAGAAAAGAGATTACAAGGATGACGACGATAAGGGTGGTGGAGGTAGTGGAGG
ΔPETase-R	CCGGAATTCCTAGATCAATAGGGCAATGGC
PETase (S160A)-F	CCGCTCGAGAAAAGAGATTACAAGGATGACG
PETase (S160A) -overlap-F	GTTATGGGTTGGGCCATGGGTGGTGG
PETase (S160A) -overlap-R	CCACCACCCATGGCCCAACCCATAAC
PETase (S160A)-R	CCGGAATTCCTAGATCAATAGGGCAATGG
p51-F	GGAATTCCATATGCAAACCAATCCTTATGCCCG
p51-R	CCGCTCGAGCTAGATCAATAGGGCAATGGCAAC
PL-F	GGAATTCCATATGCAAACCAATCCTTATGCCCG
PL-R	CCGCTCGAGACTACCACCTCCTCCACTACCTCCA
