# Supplementary material for: Biodegradation of highly crystallized poly(ethylene terephthalate) through cell surface codisplay of bacterial PETase and hydrophobin
Source: Nat Commun. 2022 Nov 21;13:7138. doi: 10.1038/s41467-022-34908-z (PMC9681837; doi:10.1038/s41467-022-34908-z)
Supplement: Supplementary file 3 — Description of Additional Supplementary Files [file 41467_2022_34908_MOESM3_ESM.pdf]

Description of Additional Supplementary Files

Supplementary Movie 1. MD simulation video of the dynamic process of PET adsorption by the HFBI displayed system.

Supplementary Movie 2. MD simulation video of the dynamic process of PET adsorption by the co-display system.
