# Supplementary material for: Biodegradation of highly crystallized poly(ethylene terephthalate) through cell surface codisplay of bacterial PETase and hydrophobin
Source: Nat Commun. 2022 Nov 21;13:7138. doi: 10.1038/s41467-022-34908-z (PMC9681837; doi:10.1038/s41467-022-34908-z)
Supplement: Supplementary file 6 — Reporting Summary [file 41467_2022_34908_MOESM6_ESM.pdf]

nature portfolio

Corresponding author(s): Yanyan Wang, Zefang Wang

Last updated by author(s): Nov 9, 2022

Reporting Summary

Nature Portfolio wishes to improve the reproducibility of the work that we publish. This form provides structure for consistency and transparency in reporting. For further information on Nature Portfolio policies, see our Editorial Policies and the Editorial Policy Checklist.

Statistics

For all statistical analyses, confirm that the following items are present in the figure legend, table legend, main text, or Methods section.

n/a	Confirmed	
☐	☒	The exact sample size (n) for each experimental group/condition, given as a discrete number and unit of measurement
☐	☒	A statement on whether measurements were taken from distinct samples or whether the same sample was measured repeatedly
☐	☒	The statistical test(s) used AND whether they are one- or two-sided<br>*Only common tests should be described solely by name; describe more complex techniques in the Methods section.*
☒	☐	A description of all covariates tested
☒	☐	A description of any assumptions or corrections, such as tests of normality and adjustment for multiple comparisons
☐	☒	A full description of the statistical parameters including central tendency (e.g. means) or other basic estimates (e.g. regression coefficient) AND variation (e.g. standard deviation) or associated estimates of uncertainty (e.g. confidence intervals)
☒	☐	For null hypothesis testing, the test statistic (e.g. F, t, r) with confidence intervals, effect sizes, degrees of freedom and P value noted<br>*Give P values as exact values whenever suitable.*
☒	☐	For Bayesian analysis, information on the choice of priors and Markov chain Monte Carlo settings
☒	☐	For hierarchical and complex designs, identification of the appropriate level for tests and full reporting of outcomes
☒	☐	Estimates of effect sizes (e.g. Cohen's d, Pearson's r), indicating how they were calculated

Our web collection on statistics for biologists contains articles on many of the points above.

Software and code

Policy information about availability of computer code

Data collection	SSRF beamline BL17U1, BL18U1 , BL19U1, BL02U1
Data analysis	HKL3000 721.3, Phenix 1.14, COOT 0.8.9, PyMol 2.0.1, UCSF Chimera X 1.2.5, SWISS-MODEL server, ImageJ 1.52a, Schrödinger2021-3

For manuscripts utilizing custom algorithms or software that are central to the research but not yet described in published literature, software must be made available to editors and reviewers. We strongly encourage code deposition in a community repository (e.g. GitHub). See the Nature Portfolio guidelines for submitting code & software for further information.

Data

Policy information about availability of data

All manuscripts must include a data availability statement. This statement should provide the following information, where applicable:

- Accession codes, unique identifiers, or web links for publicly available datasets
- A description of any restrictions on data availability
- For clinical datasets or third party data, please ensure that the statement adheres to our policy

The coordinates for crystal structures of wild-type PETase and PETase-linker have been deposited in the Protein Data Bank (PDB), with the accession codes 8GU5 [https://doi.org/10.2210/pdb8GU5/pdb] and 8GU4 [https://doi.org/10.2210/pdb8GU4/pdb], respectively. The data generated in this study are provided in the Supplementary Information and the Source Data file provided with this paper. Data is also available from the corresponding author upon request.

Field-specific reporting

Please select the one below that is the best fit for your research. If you are not sure, read the appropriate sections before making your selection.

- [x] Life sciences
- [ ] Behavioural & social sciences
- [ ] Ecological, evolutionary & environmental sciences

For a reference copy of the document with all sections, see nature.com/documents/nr-reporting-summary-flat.pdf

Life sciences study design

All studies must disclose on these points even when the disclosure is negative.

Sample size: All the experiments are repeated three times independently. Data are presented as mean values +/- SD. The sample size used in the article is calculated according to the OD600 of yeast cells.

Data exclusions: No data were excluded.

Replication: To ensure reproducibility of experimental findings, each assay was performed at least three times to confirm the results. All repeated attempts were successful.

Randomization: Animals or human research participants were not involved in this study. Randomization was not required, as all data were performed with recombinant proteins and yeast cells.

Blinding: Animals or human research participants were not involved in this study. No blinding used in this study.

Reporting for specific materials, systems and methods

We require information from authors about some types of materials, experimental systems and methods used in many studies. Here, indicate whether each material, system or method listed is relevant to your study. If you are not sure if a list item applies to your research, read the appropriate section before selecting a response.

Materials & experimental systems

n/a	Involved in the study	
[ ]	[x]	Antibodies
[x]	[ ]	Eukaryotic cell lines
[x]	[ ]	Palaeontology and archaeology
[x]	[ ]	Animals and other organisms
[x]	[ ]	Human research participants
[x]	[ ]	Clinical data
[x]	[ ]	Dual use research of concern

Methods

n/a	Involved in the study	
[x]	[ ]	ChIP-seq
[x]	[ ]	Flow cytometry
[x]	[ ]	MRI-based neuroimaging

Antibodies

Antibodies used

Antibodies we used:
1) rabbit anti-FLAG antibody (ABclonal, catalog# AE092, 1:2000),
2) goat anti-rabbit IgG antibody (SUNGENE BIOTECH, catalog# LK2001, 1:2000),
3) goat anti-mouse antibody (SUNGENE BIOTECH, catalog# LK2003, 1:2000),
4) mouse anti-His antibody (SUNGENE BIOTECH, catalog# LK8001, 1:1000),
5) primary mouse HFBI antibody (storage in our laboratory, 1:1000)
6) FITC goat anti-mouse IgG (H+L) (Abclonal, catalog# AS001, 1:200)
7) rhodamine (TRITC) goat anti-rabbit IgG (H+L) (Abclonal, catalog# AS040, 1:200)

Validation

1) Rabbit anti-FLAG antibody (ABclonal, catalog# AE092) was validated by Zhu K, Shan Z, Zhang L, Wen W. Phospho-Pon Binding-Mediated Fine-Tuning of Plk1 Activity. Structure. 2016 Jul 6;24(7):1110-9. Manufacturer's website:https://abclonal.com.cn/Datasheet/Antibodies/AE092.pdf.
2) Manufacturer's website: http://www.sungenebiotech.com/uploadfiles/pdf/Goat%20anti-Rabbit%20IgG-H+L--HRP.pdf
3) Manufacturer's website: http://www.sungenebiotech.com/uploadfiles/pdf/Goat%20anti-Mouse%20IgG-H+L--HRP.pdf
4) Manufacturer's website: http://www.sungenebiotech.com/file/20160928/20160928163753_21744.pdf
5) The primary mouse HFBI antibody was produced by our laboratory. This antibody was validated using Western Blot with purified proteins and immunohistochemistry with cultured cells using positive and negative controls.
6) FITC goat anti-mouse IgG (H+L) was validated by Xie F, Su P, Pan T, Zhou X, Li H, Huang H, Wang A, Wang F, Huang J, Yan H, Zeng L, Zhang L, Zhou F. Engineering Extracellular Vesicles Enriched with Palmitoylated ACE2 as COVID-19 Therapy. Adv Mater. 2021 Dec;33(49):e2103471. doi: 10.1002/adma.202103471. Manufacturer's website: https://abclonal.com.cn/Datasheet/Antibodies/AS001.pdf?

v=1660975258
7) rhodamine (TRITC) goat anti-rabbit IgG (H+L) was validated by Xie F, Su P, Pan T, Zhou X, Li H, Huang H, Wang A, Wang F, Huang J, Yan H, Zeng L, Zhang L, Zhou F. Engineering Extracellular Vesicles Enriched with Palmitoylated ACE2 as COVID-19 Therapy. Adv Mater. 2021 Dec;33(49):e2103471. doi: 10.1002/adma.202103471. Manufacturer's website: https://abclonal.com.cn/Datasheet/Antibodies/AS040.pdf?v=1660975268
